# Determinants of non-invasive ventilation success or failure in morbidly obese patients in acute respiratory failure

**DOI:** 10.1186/cc12080

**Published:** 2013-03-19

**Authors:** M Lemyze, P Taufour, J Mallat, O Nigeon, D Thevenin

**Affiliations:** 1 Schaffner Hospital, Lens, France

## Introduction

Malignant obesity hypoventilation syndrome (MOHS) refers to the association between morbid obesity (body mass index >40 kg/m^2^), daytime hypercapnia (PCO_2 _>45 mmHg) after other respiratory or neuromuscular causes of alveolar hypoventilation have been excluded, and multiple organ disorders (diabetes mellitus, arterial hypertension, metabolic syndrome, obstructive sleep apnea syndrome) [1]. Acute respiratory failure (ARF) is a common life-threatening complication of MHOS. Non-invasive ventilation (NIV) provides the cornerstone of the therapeutic management of decompensated MOHS [2]. We aimed to identify the determinants of NIV success or failure in this indication.

## Methods

A monocentric prospective observational study including 48 patients with MOHS and treated by NIV for ARF over a 2-year period.

## Results

NIV failed to reverse ARF in only seven patients. NIV failure was associated with a sixfold increase in in-hospital mortality (85.7% vs. 14.6%; *P <*0.001). Factors associated with NIV failure and need for endotracheal intubation included pneumonia (*n = *5, 71.4% vs. *n = *8, 19.5%; *P *= 0.01), higher SOFA (11 vs. 5; *P <*0.001) and SAPS 2 (69 vs. 39; *P *= 0.001) score at admission. The only factor associated with successful response to NIV was idiopathic decompensated MOHS (*n = *19, 100% vs. *n = *0, 0%; *P *= 0.03). In these patients, pH (7.23 vs. 7.28 vs. 7.44, respectively, at H0, H2, H24; *P <*0.05) and PCO_2 _(74.5 vs. 68.5 vs. 57 mmHg, respectively, at H0, H2, H24; *P <*0.05) dramatically improved with NIV.

## Conclusion

NIV provides a very efficient therapy to reverse hypercapnic ARF in morbidly obese patients. Multiple organ failure and pneumonia are the main factors associated with NIV failure in patients with MOHS.

## References

[B1] Marik PE J Intensive Care Med 2012 in press

[B2] Piper AJ Am J Respir Crit Care Med 2011 183 292 298 10.1164/rccm.201008-1280CI 21037018

